# Implementation of a New Discharge Card in the Department of Internal Medicine at Almanagil Teaching Hospital: A Quality Improvement Project

**DOI:** 10.7759/cureus.92710

**Published:** 2025-09-19

**Authors:** Abubakr Muhammed, Abdulrahman Abbas Yusuf Mohammed, Maali Yousif Mustafa Idris, Lujain Moqaouas, Fatima Babikir Ali Mohamed, Weaam Mohammed Awad Ahmed, Lugien Ahmed Mohamed Ibrahim, Mohammed Osman Ahmed Osman, Hind Mohamed Bashir Mohamed Ghalib, Alaa Albadwy, Mohannad Samir, Shaza Dafalla, Mohamed Abdalla Elawad Wedatalla, Salma Abdalla Elshaikh, Mohamedelmugtaba Awad Mohamed Abuzaid, Abdalmahmoud Asadig Kanan Ahmed, Elraiah Abass Yousif Eltiraifi, Ahmed Hashim Mohamad, Mustafa Awad, Mohammad Alzain Adam

**Affiliations:** 1 Radiology, University of Cape Town, Cape Town, ZAF; 2 Internal Medicine, Almanagil Teaching Hospital, Almanagil, SDN; 3 Medicine, Wad Medani Teaching Hospital, Wad Medani, SDN; 4 Internal Medicine, Al Tadawi Specialty Hospital, Dubai, ARE; 5 Internal Medicine, University of Bahri, Khartoum, SDN; 6 Molecular Medicine, Institute of Endemic Diseases, Khartoum, SDN; 7 Internal Medicine, The National Ribat University, Khartoum, SDN; 8 Internal Medicine, United Lincolnshire Hospitals NHS Trust, Lincolnshire, GBR; 9 Radiation Oncology, St Luke's Hospital, Dublin, IRL; 10 Internal Medicine, Ministry of Health and Prevention (MOHAP), Ajman, ARE; 11 Ambulatory Healthcare Services (AHS), Abu Dhabi Health Services Company (SEHA), Al Ain, ARE; 12 Internal Medicine, Sudan Medical Specialization Board, Khartoum, SDN

**Keywords:** clinical audit cycles, quality improvement project (qip), standardized discharge card, statistically significant compliance gains, two cycles

## Abstract

Background: Inadequate discharge documentation in the Internal Medicine Department of Almanagil Teaching Hospital threatened patient safety, contributing to medication errors and disrupted care. This project implemented a standardized discharge card to address major deficiencies identified by initial audits.

Methods: Using a pre-/post-intervention design (July 1 to August 1, 2025), an initial audit of 50 records allowed for the joint creation of a structured discharge card. Staff training came before the implementation period, followed by a later audit of 50 records to evaluate completeness against 52 varied parameters. Statistical significance (p<0.05) was calculated using chi-square analysis.

Results: The post-intervention period showed considerable improvement: patient identifier compliance approached complete adherence (contact details: 0% to 98%-100%), clinical documentation improved significantly (allergy status: 0% to 100%; discharge medications: 42% to 98%), and follow-up planning notably improved (follow-up date: 10% to 90%). Over 85% of parameters showed statistically significant improvements, with 32 of 52 key fields reaching compliance rates of 98% and higher.

Conclusion: Implementing standardized discharge documentation and focused staff training significantly enhanced record completeness in this resource-limited environment. While ongoing issues with complex patient histories and discharge procedures require further attention, this model provides a reproducible solution for improving care transitions in similar healthcare settings, particularly in low- and middle-income countries where standardized discharge processes are less established.

## Introduction

Effective discharge documentation is a critical component of patient care transition from hospital to community settings, ensuring continuity, safety, and quality of care after hospitalization. Incomplete or inconsistent discharge summaries can jeopardize patient outcomes by causing medication errors, delays in follow-up care, and unnecessary repetition of investigations. Recognizing these challenges, national and international health authorities have emphasized the importance of standardized, comprehensive discharge information to improve patient safety and care coordination [1-3].

The Health Information and Quality Authority (HIQA) in Ireland has developed a national standard for patient discharge summary information, which aims to enhance communication between hospital and primary care providers [1]. This standard ensures that relevant and complete health information is consistently available, reducing risks during care transitions and supporting streamlined discharge processes. Studies from various settings, including Sudanese hospitals, have demonstrated that structured discharge documentation with clear headings improves the quality and completeness of clinical information at the time of discharge [4-6].

The Department of Internal Medicine at Almanagil Teaching Hospital, serving a significant population in Sudan, has recognized the need to improve its discharge documentation system. This quality improvement project focuses on the implementation of a new discharge card designed to standardize and enhance the discharge process by providing structured, comprehensive, and clear information to healthcare providers. Inspired by successful initiatives at similar institutions and aligned with best practices endorsed by global healthcare frameworks, this project was undertaken with the following objectives: to design and implement a standardized discharge card, to evaluate its impact on the completeness and accuracy of discharge documentation, and to assess staff compliance after its introduction [6-8].

## Materials and methods

Study design and setting

This quality improvement project (QIP) was conducted at Almanagil Teaching Hospital, a tertiary health institution in Sudan that serves a heterogeneous population of both urban and rural residents. The project was focused on the Department of Internal Medicine, which had been identified as the area needing improvement in the discharge documentation process. The QIP was initiated on July 1, 2025, and is still ongoing, having completed the first stage of intervention and assessment as of August 1, 2025.

Study phases and interventions

First Cycle (Pre-intervention State and Root Cause Analysis) (July 1 to July 11)

The initial audit identified significant deficiencies in the discharge documentation process at Almanagil Teaching Hospital. A total of 50 discharge cards from the Department of Internal Medicine were manually reviewed during the first cycle to establish baseline practices. At that time, discharge summaries were prepared in handwritten format, as no electronic or computerized discharge system was in place. The audit revealed frequent omissions of critical patient information, such as date of birth, contact numbers, and follow-up instructions, alongside the absence of a standardized discharge card. To better understand the underlying issues, a root cause analysis was conducted. This involved a detailed review of the existing handwritten records and consultations with physicians, nurses, and administrative staff to gather their insights. The findings confirmed systemic workflow gaps, inconsistent practices, and limited staff awareness of documentation requirements. These results were subsequently used to guide the design of a standardized discharge template and outline targeted training strategies for the next phase of the audit.

Interventions (July 12 to July 21)

A standardized discharge card was co‑developed by the quality improvement team, medical staff, and hospital management. It incorporated comprehensive fields for patient demographics (name, age, gender, date of birth, address, and contact number), clinical information (presenting complaint, diagnosis, stage of disease, allergies, investigation results, treatment provided, discharge medications, and follow‑up plan), and professional details (name, role, date/time, and signature). A copy of the standardized discharge card template is provided in the Appendices.

Targeted training sessions were delivered to all clinical staff in the Department of Internal Medicine, emphasizing the role of complete and accurate documentation in patient safety and continuity of care. These sessions consisted of short interactive lectures, practical demonstrations on how to complete the standardized discharge card, and group discussions to address staff questions. Feedback from physicians and nurses was positive, and their suggestions were incorporated to refine minor aspects of the card before the second audit cycle.

The new format was implemented and actively monitored by the audit team through daily reviews, with feedback collected from staff to resolve challenges and ensure high compliance.

Second Cycle (Post-intervention Evaluation) (July 22 to August 1)

Following the first cycle, a new standardized discharge card template was developed, incorporating all essential patient, clinical, and follow‑up information fields. Comprehensive training sessions were provided to medical staff in the Department of Internal Medicine to ensure consistent usage. The second audit cycle, which reviewed 50 discharge cards, showed a transformative improvement in documentation quality, with most parameters reaching or nearing full compliance. Notable gains included demographic details such as gender (2% to 100%), contact information (0% to ≥98%), complete referral details (≤44% to ≥98%), and full admission/discharge records (≤8% to 100%). Clinical fields such as diagnosis, stage of condition, investigation results, allergy status, and discharge medications all improved to ≥98% compliance. Follow-up details also recorded major gains (6%-10% to 73%-90%). Despite these advances, certain areas, such as consent form documentation, discharge against medical advice (DAMA) details, and readmission risk assessment, remained low. Overall, the intervention demonstrated that standardized tools combined with staff engagement can significantly enhance discharge documentation completeness and accuracy.

Data analysis

Discharge cards were randomly selected and evaluated by three physicians trained in standardized assessment protocols to ensure consistency. Documentation completeness and adherence to updated standards were analyzed using quantitative and qualitative methods, supplemented by stakeholder feedback. While randomization and trained reviewers strengthened reliability, the absence of documented blinding during evaluations introduced potential bias, a limitation affecting interpretation. The findings provided clinically valid insights for targeted improvements; however, future studies should incorporate blinding to further reduce bias.

Evaluation

Feedback and Effectiveness Assessment

The impact of the intervention was assessed by comparing documentation completeness and compliance rates before and after the introduction of the standardized discharge card. Structured feedback from healthcare providers and patients was used to identify ongoing challenges and inform future improvements.

Ethical considerations

Ethical and managerial approval for the QIP was obtained from Almanagil Teaching Hospital (approval number: 25-QIP-008). All personal and clinical data were anonymized to protect patient privacy and confidentiality. Future projects should address the study's failure to explicitly document participants' informed consent to ensure comprehensive ethical compliance.

## Results

A total of 100 patient records were reviewed, comprising 50 from the first audit cycle and 50 from the second. Overall, the introduction of the standardized discharge card and staff training led to substantial improvements in the completeness of documentation across almost all assessed domains (Table 1).

**Table 1:** Progressive enhancements in discharge card documentation across two consecutive cycles Documentation compliance across key parameters before (first cycle) and after (second cycle) implementation of a standardized discharge card (n=50 per cycle) in the Department of Internal Medicine at Almanagil Teaching Hospital. Values are presented as counts and percentages. The table includes the improvement summary for each parameter, the p-value calculated using the chi-square test, and notes on statistical significance. A p-value < 0.05 was considered statistically significant. Parameters are grouped under domains: patient demographics, referrer details, admission details, discharge details, clinical information, allergy documentation, medications and professional documentation, medical history and physical examination, treatment and outcomes, follow-up and continuity of care, and discharge process. "New field" indicates items that were absent in the first cycle and introduced during the intervention. DAMA: discharge against medical advice

Domain	Parameter	First cycle (n=50)	Second cycle (n=50)	Improvement summary	p-value (chi-square)
Patient demographics	Name recorded	50 (100%)	50 (100%)	No change, remained at 100%	1.000
	Age specified	45 (90%)	50 (100%)	+1.1×	0.022
	Gender indicated	1 (2%)	50 (100%)	+50×	<0.001
	Telephone number listed	0 (0%)	49 (98%)	New field; 98% compliance	<0.001
	Hospital file number noted	11 (22%)	50 (100%)	+4.5×	<0.001
	Address included	0 (0%)	50 (100%)	New field; 100% compliance	<0.001
	Self-referral status indicated	0 (0%)	50 (100%)	New field; 100% compliance	<0.001
Referrer details	Referrer's name	4 (8%)	50 (100%)	+12.5×	<0.001
	Referrer's role	2 (4%)	50 (100%)	+25×	<0.001
	Referrer's organization	22 (44%)	50 (100%)	+2.3×	<0.001
	Referrer's contact details	0 (0%)	49 (98%)	New field; 98% compliance	<0.001
Admission details	Admitting unit	2 (4%)	50 (100%)	+25×	<0.001
	Other admission details	20 (40%)	50 (100%)	+2.5×	<0.001
	Type of admission	0 (0%)	50 (100%)	New field; 100% compliance	<0.001
	Admission date	0 (0%)	50 (100%)	New field; 100% compliance	<0.001
Discharge details	Discharging consultant	2 (4%)	50 (100%)	+25×	<0.001
	Discharging unit	1 (2%)	50 (100%)	+50×	<0.001
	Discharge destination	3 (6%)	50 (100%)	+16.7×	<0.001
	Discharge address (if different)	0 (0%)	50 (100%)	New field; 100% compliance	<0.001
	Discharge date	23 (46%)	50 (100%)	+2.2×	<0.001
	Patient's condition at discharge	3 (6%)	50 (100%)	+16.7×	<0.001
	Other discharge details	16 (32%)	49 (98%)	+3.1×	<0.001
Clinical information	Diagnosis entered	44 (88%)	50 (100%)	+1.1×	0.012
	Stage of condition	1 (2%)	50 (100%)	+50×	<0.001
	Clinical summary	24 (48%)	50 (100%)	+2.1×	<0.001
	Investigation results	17 (34%)	50 (100%)	+2.9×	<0.001
	Additional comments	0 (0%)	49 (98%)	New field; 98% compliance	<0.001
	Special remarks	0 (0%)	50 (100%)	New field; 100% compliance	<0.001
Allergy documentation	Allergy status	0 (0%)	50 (100%)	New field; 100% compliance	<0.001
	Allergens recorded	0 (0%)	50 (100%)	New field; 100% compliance	<0.001
	Reactions recorded	0 (0%)	49 (98%)	New field; 98% compliance	<0.001
	Severity recorded	0 (0%)	49 (98%)	New field; 98% compliance	<0.001
Medications and documentation	Discharge medication/device list	21 (42%)	49 (98%)	+2.3×	<0.001
	Professional's name	7 (14%)	50 (100%)	+7.1×	<0.001
	Professional's role	5 (10%)	50 (100%)	+10×	<0.001
	Date and time	3 (6%)	50 (100%)	+16.7×	<0.001
	Signature	0 (0%)	49 (98%)	New field; 98% compliance	<0.001
Medical history and examination	Presenting complaint	39 (78%)	50 (100%)	+1.3×	<0.001
	Past medical history	24 (48%)	41 (81.6%)	+1.7×	<0.001
	Surgical history	1 (2%)	12 (24.5%)	+12.2×	0.001
	Social history	0 (0%)	14 (28.6%)	New field; 28.6% compliance	<0.001
	Family history	0 (0%)	8 (16.3%)	New field; 16.3% compliance	0.005
	Physical examination	12 (24%)	29 (57.1%)	+2.4×	0.001
Treatment and outcomes	Treatment given	29 (58%)	30 (59.2%)	+1.0×	0.910
	Response to treatment	1 (2%)	25 (49%)	+24.5×	<0.001
	Complications during stay	0 (0%)	26 (51%)	New field; 51% compliance	<0.001
	Discontinued medications	0 (0%)	3 (6.1%)	New field; 6.1% compliance	0.096
	Discontinued medications and reasons	0 (0%)	3 (6.1%)	New field; 6.1% compliance	0.096
	Long-term medication status	9 (18%)	17 (34.7%)	+1.9×	0.050
	Long-term medication list	3 (6%)	17 (34.7%)	+5.8×	<0.001
	Interactions/warnings	0 (0%)	9 (18.4%)	New field; 18.4% compliance	0.003
Follow-up and continuity of care	Follow-up required	4 (8%)	43 (85.7%)	+10.7×	<0.001
	Follow-up date	5 (10%)	45 (89.8%)	+9×	<0.001
	Follow-up location/clinic	3 (6%)	45 (89.8%)	+15×	<0.001
	Doctor to see	1 (2%)	37 (73.5%)	+36.8×	<0.001
	Home/community care needed	0 (0%)	3 (6.1%)	New field; 6.1% compliance	0.096
	Home/community care specified	0 (0%)	3 (6.1%)	New field; 6.1% compliance	0.096
	Patient/family education provided	0 (0%)	32 (63.3%)	New field; 63.3% compliance	<0.001
	Topics of education	0 (0%)	28 (55.1%)	New field; 55.1% compliance	<0.001
	Medical report required	1 (2%)	19 (38.8%)	+19.4×	<0.001
Discharge process	Summary shared with referrer	1 (2%)	26 (51%)	+25.5×	<0.001
	Method of sending summary	1 (2%)	26 (51%)	+25.5×	<0.001
	Staff handing over recorded	0 (0%)	27 (53.1%)	New field; 53.1% compliance	<0.001
	Risks discussed with patient/family	0 (0%)	27 (53.1%)	New field; 53.1% compliance	<0.001
	List of discussed risks	0 (0%)	19 (38.8%)	New field; 38.8% compliance	<0.001
	DAMA documented	0 (0%)	1 (2%)	New field; 2% compliance	0.480
	Reason/signatory for DAMA	0 (0%)	0 (0%)	No documentation	N/A
	Consent form signed	0 (0%)	0 (0%)	No documentation	N/A
	Consent given by	0 (0%)	1 (2%)	New field; 2% compliance	0.480
	Mode of transport	0 (0%)	26 (51%)	New field; 51% compliance	<0.001
	Time of discharge	4 (8%)	26 (51%)	+6.4×	<0.001
	Length of stay	0 (0%)	26 (51%)	New field; 51% compliance	<0.001

Patient demographics showed marked enhancement, with significant increases in the recording of age, gender, contact information, and hospital file numbers. Referrer details and admission information, which were poorly documented at baseline, reached near-universal compliance post-intervention.

Discharge-related documentation also improved considerably. Key elements such as discharge consultant, unit, destination, date, and condition at discharge achieved almost complete compliance, while additional discharge details similarly demonstrated strong gains. The recording of follow-up dates, which was only five (10%) at baseline, increased substantially to 45 (89.8%) in the second cycle, highlighting the effectiveness of the standardized discharge card in ensuring continuity of care.

Clinical information, including primary and secondary diagnoses, clinical summaries, investigation results, and special remarks, was more consistently captured in the second cycle. Allergy documentation, which was completely absent in the first cycle, showed near-total completion after the intervention.

Medication records and professional accountability measures improved substantially, with discharge medication lists, signatures, and professional details recorded in nearly all cases post-intervention. Documentation of medical history and physical examinations also improved, although surgical, social, and family histories remained less frequently recorded compared to other fields.

Treatment and outcome data showed significant progress in documenting responses to treatment, complications, and long-term medications, although areas such as discontinued medications and interactions were less well addressed.

Follow-up and continuity of care parameters displayed notable improvement, particularly in recording follow-up requirements, dates, clinic locations, and responsible physicians. Patient and family education was increasingly documented, although home or community care needs remained poorly captured.

The discharge process domain also demonstrated progress, with greater attention to sharing discharge summaries, documenting handovers, and discussing risks. Nonetheless, some areas, such as consent forms, DAMA documentation, and readmission risk assessments, remained unrecorded in both cycles.

Overall, more than 85% of the assessed parameters showed statistically significant improvement between the two audit cycles, with most achieving near-total compliance after the intervention (Table 1).

## Discussion

This project was designed to address the long-standing gaps in discharge documentation by introducing a structured discharge card within the Department of Internal Medicine at Almanagil Teaching Hospital. The period surrounding hospital discharge is considered one of the most fragile stages in patient management, as inadequate or inaccurate communication at this point can compromise safety and continuity of care. Missing or unclear information may delay follow-up, lead to the omission of essential therapies, or cause patients to undergo unnecessary repeat investigations and treatments. Evidence from prior studies has shown that close to 20% of patients encounter adverse events in the period immediately after discharge, and a considerable proportion of these complications are thought to be avoidable [9,10].

The current quality improvement project demonstrated substantial enhancement in the completeness and accuracy of discharge documentation within the Department of Internal Medicine at Almanagil Teaching Hospital following the introduction of a standardized discharge card coupled with targeted staff training. This intervention resulted in near-complete improvements across almost all measured parameters, with the majority exceeding 98% compliance in the post-intervention assessment, in alignment with findings from comparable institutions [4,6]. The most pronounced improvements were observed in critical areas impacting patient safety, including patient identification and administrative information (e.g., contact details increased from 0% to 98%, p<0.001), allergy documentation (from 0% to 100%, p<0.001), discharge medication lists (from 42% to 98%, p<0.001), and follow-up planning (follow-up dates improved from 10% to 90%, p<0.001), along with enhanced professional accountability key components previously marked by poor documentation [1,5,8,11].

These results strongly indicate that the initial deficiencies were primarily systemic rather than individual, arising from the absence of a standardized template and significant variability in practice [5,6]. The newly implemented card incorporated clear prompts and explicit fields, effectively reducing ambiguity and ensuring uniform documentation of essential information, in accordance with HIQA guidelines [1] and best practices highlighted in related studies [4,7].

A recent clinical audit conducted at Dongola Specialized Hospital, where a standardized discharge card was implemented over two cycles, similarly demonstrated substantial improvements in discharge documentation quality [4]. At Dongola, complete patient information increased from 66% in the first cycle to 100% in the second cycle, clinical summaries improved from 56% to 96%, discharge plans from 60% to 90%, and accurate medication lists rose to 88% post-intervention. Furthermore, preliminary data indicated a 15% reduction in readmission rates, which illustrates the possible clinical benefits of enhanced documentation [4]. Both audits underscore that structured discharge tools, combined with targeted staff training, effectively address previously systemic gaps, particularly in areas crucial for patient safety, continuity of care, and professional accountability [4]. In line with these findings, Tan et al. reported that although 93.1% of discharge summaries contained an accurate medication list, only 50% of medication changes included details on indication and outpatient management, underscoring that while medication documentation often improves, contextual details such as rationale and follow-up remain under-documented [12].

The project has highlighted specific areas that require increased attention. The recording of extensive histories, including past medical, surgical, social, and family issues, and specific treatment outcomes such as patient response, complications, and drug interactions, has improved but remains inadequate, reflecting the acknowledged difficulties inherent in extensive clinical documentation research [11]. At the same time, despite significant improvements in other aspects of the discharge process itself (e.g., sharing summaries with referrers increased from 2% to 51%, p<0.001), compliance with near-optimal standards established in less complex specialist areas was not matched. Notably, aspects such as consent forms, DAMA information, readmission risk assessment, and community care requirements were poorly documented. These elements entail complex processes that extend significantly beyond simple form filling and may require additional approaches, such as standardized handover processes, checklists, and embedded risk assessment tools [1,7]. Comparable limitations have been noted even in electronic systems; Shehzad et al. observed that although over 80% of electronic discharge letters included discharge medications and follow-up actions, documentation of allergy status and investigations was limited to 27% and 49%, respectively, showing that challenges in achieving comprehensive documentation persist across both electronic and paper-based systems [13].

Moreover, Claassen et al. highlighted the value of telephone-based follow-up procedures in minimizing missing data in longitudinal studies, demonstrating that such strategies significantly improved data retention rates [14]. Applying similar approaches within discharge documentation processes could provide an additional safeguard against incomplete records, particularly in resource-limited settings where manual documentation remains predominant. Integrating structured telephone follow-up or remote verification may thus complement the standardized discharge card, further enhancing the accuracy and continuity of patient care.

The sturdiness of the project can be attributed to its well-defined pre-/post-design, rigorous quantitative assessment, collaborative intervention development across stakeholders [4,6], and incorporation of varied training components. The large-scale and statistically significant improvements observed provide strong evidence of the intervention's success [4-6]. To ensure sustained benefits, ongoing surveillance and incorporation of the standardized discharge card into routine clinical practice are recommended [4,6].

Multiple studies have demonstrated that the use of structured discharge tools such as standardized checklists or evidence-based templates can significantly reduce readmission rates and enhance the overall safety of care transitions. For example, a study by Marano et al. found that implementing a standardized discharge checklist in patients hospitalized for heart failure resulted in a notable 12.4% absolute reduction in 30-day all-cause readmission (29.9% versus 17.5%, p=0.02) [15]. This evidence supports the addition of such structured approaches to further bolster the effectiveness of interventions like the discharge card, notably by bridging the gap between acute care documentation and post-discharge continuity.

Limitations

This study has several limitations. First, the post-intervention assessment period was relatively short, which may not adequately capture the long-term sustainability of improved documentation practices. Second, as the study was conducted at a single center, the findings may have limited generalizability to other hospitals or departments with different patient populations, resources, and workflows. Third, the audit relied on manual chart reviews, which introduces the possibility of measurement bias, as reviewers were not blinded and some information may have been inadvertently missed or misclassified. Fourth, certain aspects of patient consent and engagement were not systematically documented, restricting evaluation of the ethical and procedural completeness of the intervention. Fifth, although the intervention was generally well-received, some physicians initially expressed concern about the potential increase in workload. While familiarity with the standardized card eventually improved acceptance, the issue of workload remains an important consideration for long-term sustainability, particularly in resource-limited settings. Finally, while the study demonstrated significant improvements in the quality of discharge documentation, it did not assess downstream clinical outcomes such as medication errors, patient adherence, or long-term readmission rates, which remain important areas for future investigation.

## Conclusions

The introduction of a standard discharge card, alongside staff education programs, has greatly enhanced the quality of documentation in the Department of Internal Medicine. Key features of patient identifiers, allergies, discharge medications, and follow-up plans have achieved near-universal rates of compliance (≥98%). The result indicates that well-designed forms can compensate for systemic gaps in documentation in resource-limited settings, establishing a foundation for enhanced continuity of care. While issues remain in the detailed documentation of complex medical histories and discharges, the initiative represents a major step forward. Avenues for future work should be directed at sustaining such gains, correcting remaining discrepancies, and assessing effects on clinical outcomes. The model provides a template for similar healthcare facilities.

## Figures and Tables

**Figure 1:**
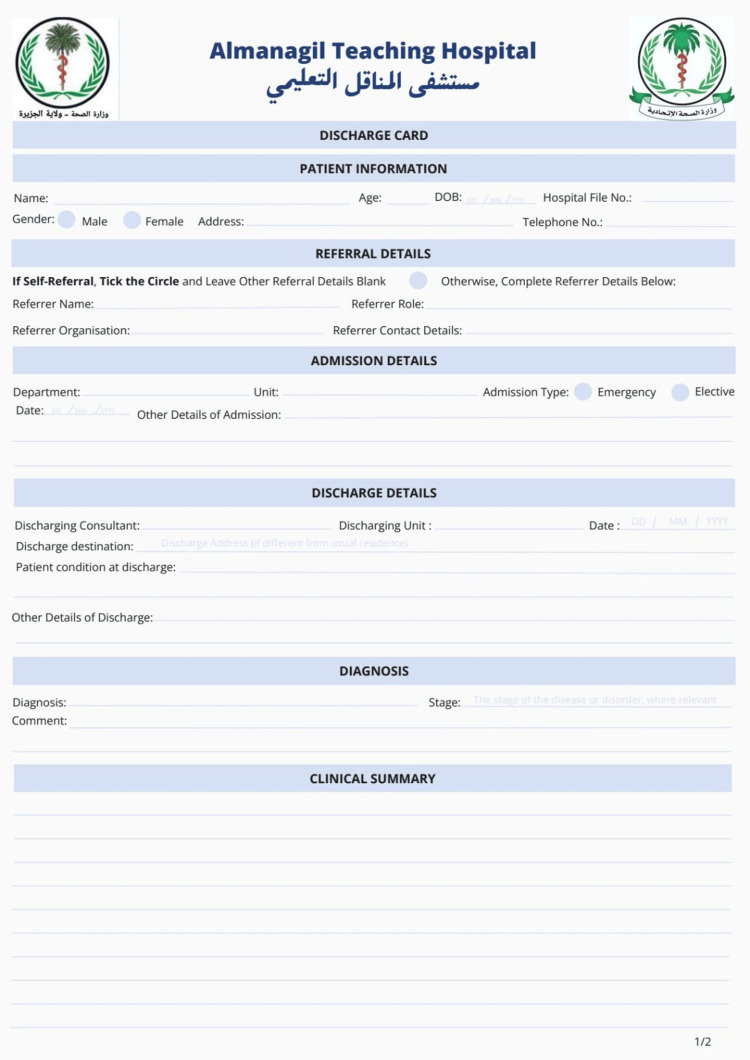
Front page of the new discharge card DOB: date of birth

**Figure 2:**
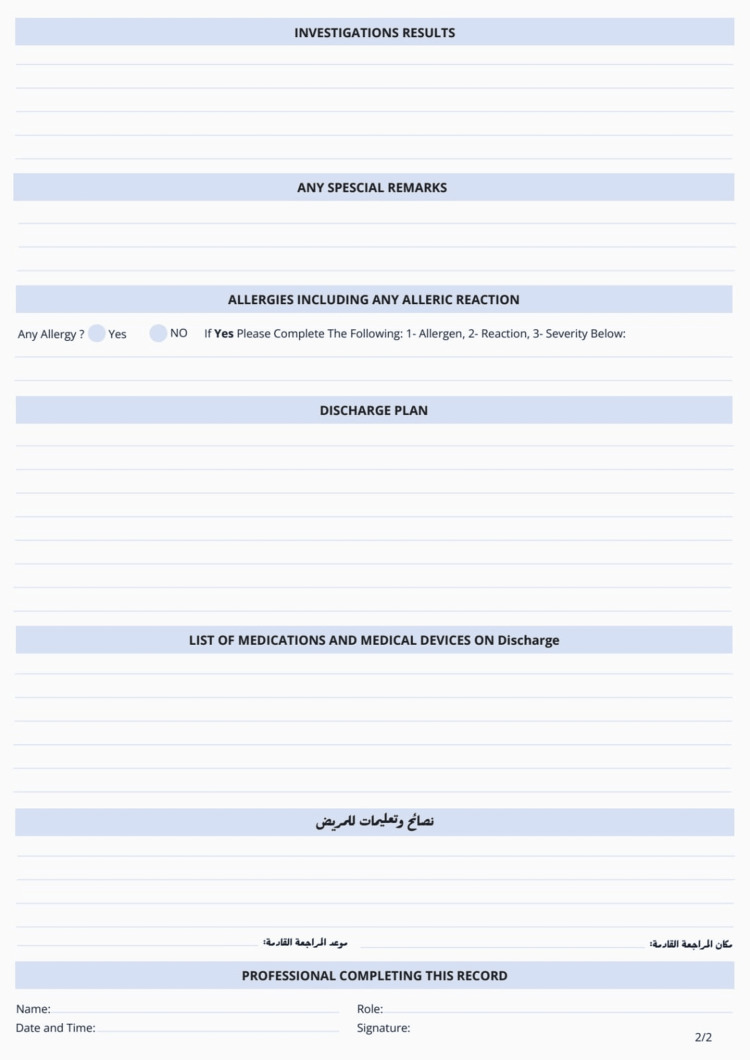
Back page of the new discharge card

## Appendices

To facilitate consistent documentation, a standardized discharge card was developed and introduced during the intervention phase. This card incorporated comprehensive fields for patient identifiers, clinical details, treatment information, and follow-up planning. A copy of the standardized discharge card template is provided in Figure 1 and Figure 2.
